# Clonal Dissemination, Emergence of Mutator Lineages and Antibiotic Resistance Evolution in *Pseudomonas aeruginosa* Cystic Fibrosis Chronic Lung Infection

**DOI:** 10.1371/journal.pone.0071001

**Published:** 2013-08-12

**Authors:** Carla López-Causapé, Estrella Rojo-Molinero, Xavier Mulet, Gabriel Cabot, Bartolomé Moyà, Joan Figuerola, Bernat Togores, José L. Pérez, Antonio Oliver

**Affiliations:** 1 Servicio de Microbiología y Unidad de Investigación, Hospital Universitario Son Espases, Palma de Mallorca, Spain; 2 Servicio de Pediatría, Hospital Universitario Son Espases, Palma de Mallorca, Spain; 3 Servicio de Neumología, Hospital Universitario Son Espases, Palma de Mallorca, Spain; University of Duisburg-Essen, Germany

## Abstract

Chronic respiratory infection by *Pseudomonas aeruginosa* is a major cause of mortality in cystic fibrosis (CF). We investigated the interplay between three key microbiological aspects of these infections: the occurrence of transmissible and persistent strains, the emergence of variants with enhanced mutation rates (mutators) and the evolution of antibiotic resistance. For this purpose, 10 sequential isolates, covering up to an 8-year period, from each of 10 CF patients were studied. As anticipated, resistance significantly accumulated overtime, and occurred more frequently among mutator variants detected in 6 of the patients. Nevertheless, highest resistance was documented for the nonmutator CF epidemic strain LES-1 (ST-146) detected for the first time in Spain. A correlation between resistance profiles and resistance mechanisms evaluated [efflux pump (*mexB, mexD, mexF*, and *mexY*) and *ampC* overexpression and OprD production] was not always obvious and hypersusceptibility to certain antibiotics (such as aztreonam or meropenem) was frequently observed. The analysis of whole genome macrorestriction fragments through Pulsed-Field Gel Electrophoresis (PFGE) revealed that a single genotype (clone FQSE-A) produced persistent infections in 4 of the patients. Multilocus Sequence typing (MLST) identified clone FQSE-A as the CF epidemic clone ST-274, but striking discrepancies between PFGE and MLST profiles were evidenced. While PFGE macrorestriction patterns remained stable, a new sequence type (ST-1089) was detected in two of the patients, differing from ST-274 by only two point mutations in two of the genes, each leading to a nonpreviously described allele. Moreover, detailed genetic analyses revealed that the new ST-1089 is a *mutS* deficient mutator lineage that evolved from the epidemic strain ST-274, acquired specific resistance mechanisms, and underwent further interpatient spread. Thus, presented results provide the first evidence of interpatient dissemination of mutator lineages and denote their potential for unexpected short-term sequence type evolution, illustrating the complexity of *P. aeruginosa* population biology in CF.

## Introduction

Chronic respiratory infection by *Pseudomonas aeruginosa* is a major driver of morbidity and mortality in cystic fibrosis patients [1], [2]. Traditionally, initial colonization is considered to be produced by unique strains acquired from environmental sources that undergo an extensive adaptation within the patient’s lungs, leading to life-long persistent infections with little patient to patient transmission [3]. The establishment of the characteristic biofilm structures and the acquisition of a plethora of adaptive mutations (leading to enhanced antimicrobial and host defenses resistance, specific metabolic adaptation and an adapted virulence), are the main responsible for the persistence of these infections despite extensive antimicrobial therapy [4]–[9].

One of the hallmarks of *P. aeruginosa* chronic respiratory infections, in contrast to acute processes, is the high prevalence of hypermutable (or mutator) strains [10]–[13]. These variants are found in 30 to 60% of CF patients and show up to 1000-fold increased spontaneous mutation rates caused by defective DNA repair pathways. Among them, the mismatch repair (MMR) system is the one most frequently affected, due to mutations in *mutS* or *mutL* genes [14]–[16]. Indeed, mutator variants are positively selected during the establishment of chronic infections, linked to the acquisition of mutations related to antibiotic resistance, biofilm growth, metabolic adaptation, or acute virulence attenuation [10], [16]–[20].

Additionally, there is growing evidence suggesting that adaptation to the CF lung environment may escape from the scale of the individual patients [21]. Indeed, the existence of concerning epidemic strains, such as the Liverpool Epidemic Strain (LES-1), capable of infecting hundreds of CF patients in different geographical locations, has been well documented for over two decades [22], [23]. Mutator variants have also been detected in a small proportion of isolates from patients infected by the LES-1 epidemic strain [24], [25], but interpatient spread of mutator variants has never been demonstrated. Moreover, recent whole genome sequence analyses have revealed that the origination of CF adapted epidemic strains may result from a limited number of specific mutations with pleiotropic effects [26].

Although whole genome sequencing and microarray analysis will soon take the lead, the current gold standard for typing *P. aeruginosa* strains with the purpose of investigating patient to patient transmission is still the analysis of whole genome macrorestriction fragments through Pulsed-Field Gel Electrophoresis (PFGE) [27]. However, the instability of PFGE profiles, mainly consequence of frequent genome rearrangements, makes this procedure unsuitable for long-term and global epidemiology studies [28]. On the other hand, Multilocus Sequence Typing (MLST), based on sequencing of 7 house keeping genes, provides a much more stable genetic signature and is still currently considered the gold standard for global epidemiology and population structure analyses [29].

In this work, we investigated the interplay between the three above described key microbiological aspects of *P. aeruginosa* CF chronic lung infections: the occurrence of transmissible and persistent strains (PFGE-MLST clonal epidemiology), the emergence of variants with enhanced mutation rates (mutators) and the evolution of antibiotic resistance.

## Results and Discussion

### Long-term Clonal Epidemiology of *P. aeruginosa* in CF: Transmissible and Persistent Strains

**Figure 1:**
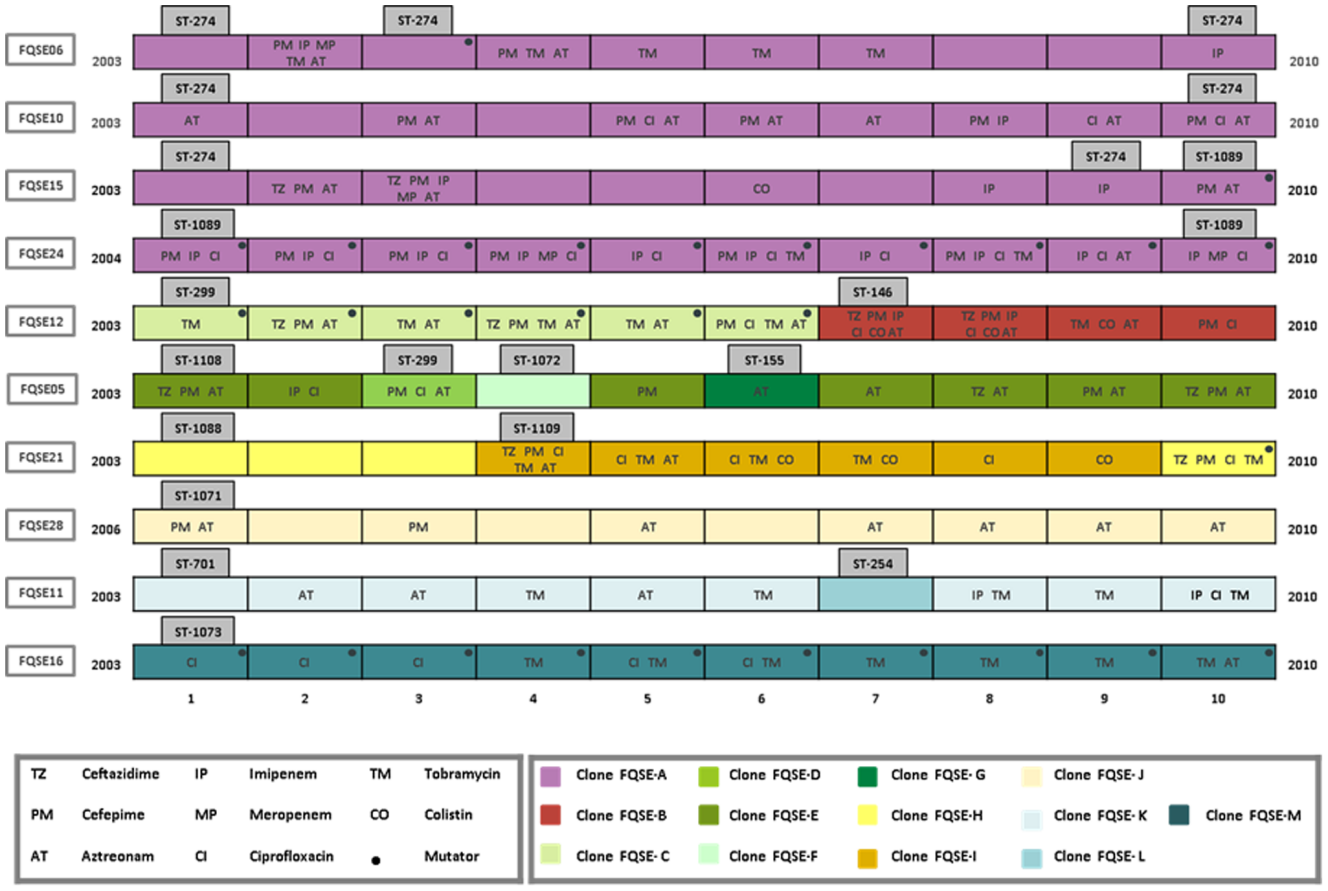
Schematic representation of the 10 sequential isolates from each of the 10 CF patients in the time frame of the study period. Each different clone is represented by a different colour. Resistance profiles and presence mutator phenotypes are indicated for each isolate. Results of MLST analysis are also provided for specific isolates.

A total of 100 *P. aeruginosa* isolates were studied, including 10 sequential isolates from each of 10 CF patients attended at the reference hospital of the Balearic Islands, Spain. Each of the sequential isolates included were separated by at least a 6-month interval, covering up to an 8-year period from 2003 to 2010. PFGE analysis revealed the presence of 13 different clones; one of them (clone FQSE-A) was detected in four patients while the other twelve were detected in single patients. Figure 1 shows the distribution of the different clones among the different patients along the 8-year study period. Six patients, including the 4 colonized by clone FQSE-A, showed a single clone over the study period, while the other 4 showed the coexistence of several (2 to 4) clones or clonal replacements (Figure 1). Therefore, results so far suggested that clone FQSE-A is a CF adapted (transmissible and persistent) strain. The epidemiological setting driving (direct or indirect) interpatient transmission is uncertain, since recommendations on segregation of patients colonized by *P. aeruginosa* from those free of this pathogen are followed in all hospital visits.

### Discrepant MLST vs PFGE Results: Role of Mutators

The first isolate per patient and clone were further analyzed by MLST, yielding 13 sequence types (ST) not entirely coincident with the clones identified by PFGE. The allelic profiles and relevant features of the 13 STs identified are shown in Table 1. Not surprisingly due to the overall higher discriminatory power (or lower stability) of PFGE compared to MLST [28]–[30], two different PFGE clones (FQSE-C and FQSE-D) shared the same ST (ST-299). Much more intriguingly, the disseminated clone FQSE-A yielded two different STs (Table 1, Figure 1). Clone FQSE-A from 3 of the 4 patients was identified as ST-274, previously detected in multiple CF patients from France, Austria and Australia according to the MLST database (http://pubmlst.org/paeruginosa/). Moreover, this clone has been simultaneously detected in several patients from another CF Unit in Madrid [31] and in a few cases of hospital-acquired infections in recent Spanish multicentre studies [32], [33]. Thus, our results add further evidence pointing out that ST-274 should be added to the growing list of CF epidemic clones [22], [29]. In contrast, clone FQSE-A from the fourth patient was identified as a new ST (ST-1089) differing from ST-274 by only two point mutations in two of the genes (*acsA* and *guaA*) each leading to a nonpreviously described allele (the only two novel alleles found in the complete collection). Additionally, in contrast to ST-274, ST-1089 showed a mutator phenotype (Table 1). Therefore, the available data clearly suggested that ST-1089 has recently evolved from the CF epidemic clone ST-274 through point mutations linked to the emergence of a mutator lineage. As expected, due to the mutational spectra of DNA MMR deficient strains [12], both mutations were G to A transversions. Moreover, both mutations were apparently not neutral, since they lead to nonsynonymous substitutions in the acetyl-coenzyme A synthetase (G435D) and the GMP synthase (G312S), which are key metabolic enzymes. Whether these mutations were positively selected because the play a role in the intense metabolic adaptation to the CF chronic lung infection setting [18] remains to be explored.

**Table 1:** Allelic profiles and relevant features of the different ST detected.

Clone	Sequence type	Allelic profile^a^							Relevant features
		*acsA*	*aroE*	*guaA*	*mutL*	*nuoD*	*ppsA*	*trpE*	
FQSE-A	ST-274	23	5	11	7	1	12	7	Detected in CF patients in Australia, Austria and France
FQSE-A	ST-1089	66	5	101	7	1	12	7	New Sequence Type, DLV of ST-274 (Mutator)
FQSE-B	ST-146	6	5	11	3	4	23	1	MDR Liverpool Epidemic Strain (LES-1)
FQSE-C/D	ST-299	17	5	36	3	3	7	3	Detected in CF patients in Australia
FQSE-E	ST-1108	6	3	17	7	3	4	7	New Sequence Type
FQSE-F	ST-1072	5	13	25	6	1	7	3	New Sequence Type
FQSE-G	ST-155	28	5	36	3	3	13	7	Detected in CF patients in Australia, Canada and France
FQSE-H	ST-1088	36	27	28	3	4	13	1	New Secuence Type
FQSE-I	ST-1109	16	14	3	11	1	15	1	New Sequence Type
FQSE-J	ST-1071	5	3	57	6	1	33	42	New Sqcuence Type
FQSE-K	ST-701	29	1	9	13	1	6	23	New Sequence Type
FQSE-L	ST-254	6	5	58	11	3	4	37	Detected in CF patients in Australia, Canada
FQSE-M	ST-1073	28	5	36	3	4	10	95	New Sequence Type (mutator)

a Nonpreviously described alleles are shown in bold.

Consistently with our findings, while mutators series appeared to be no more variable in their MLST haplotypes than nonmutator series in a previous study, the only novel alleles found were also from patients with mutator strains [34]. Moreover, a recent study has reported discrepant MLST vs PFGE results directly linked to the emergence of a mutator phenotype caused by *mutL* mutations [35], stressing the point that this gene lacks the neutrality required for an appropriate MLST marker, since MMR deficient mutators (*mutS* and *mutL*) are positively selected in CF chronic infection [15]. Likewise, a recent work has reported a strain that was not typable by MLST due to the presence of a deletion in the *mutL* fragment analyzed [30]. All together, these results indicate that frequent mutator variants from chronic infection may determine a lower stability of the MLST profiles than expected (leading to discrepant results compared to PFGE) both directly (*mutL* inactivating mutations within the gene fragment evaluated in MLST analysis) and indirectly through the increased spontaneous mutagenesis (facilitating the emergence of novel alleles through point mutations in any of the 7 genes evaluated in MLST analysis).

Regarding the other STs detected, as expected from the frequent acquisition of unique clones by CF patients from environmental sources [3], [36], 8 of the 13 MLST clones detected corresponded to nonpreviously described STs, each found in single patients (Table 1). Nevertheless, in addition to the above described clone FQSE-A/ST-274 CF epidemic strain, clonal replacement (of a MDR mutator strain) by the MDR LES-1 epidemic strain ST-146 [29], [37] was documented in one of the patients, alerting of the first detection of the likely more world-wide concerning CF epidemic clone in Spain. Although the epidemiological driver of LES-1 colonization was not specifically investigated, the fact that the patient has family links with a northern European country could help to explain the acquisition of this clone not previously detected in Spain.

### Evidence for Interpatient Spread of a Mutator Lineage (ST-1089) Evolved from a CF Epidemic Strain (ST-274)

Since ST-274 and ST-1089 show the same PFGE pattern and MLST was initially performed only in the first isolate per patient and clone, MLST analysis was extended to the last available isolate from each patient colonized by clone FQSE-A as well as the two additional sporadic mutator isolates detected in two of the patients colonized by this clone (Figure 1). In all cases, the MLST profiles coincided with that of the first isolate, except for the mutator lineage emerging from one of the patients colonized by ST-274 that was also identified as ST-1089 (Figure 1). Thus, the mutator lineage ST-1089 was detected from the first to the last isolate analyzed in one of the patients and only in the last isolate (the only one with mutator phenotype) from a second one. Nevertheless, extended analysis of available isolates of this patient from 2010 to 2012 confirmed the persistence of the ST-1089 mutator lineage. On the other hand, the sporadic mutator lineage detected in the third patient was still ST-274. Therefore, mutator lineages were detected in 3 of the 4 patients with clone FQSE-A, two belonging to ST-1089 and one to ST-274. In order to evaluate the genetic basis of hypermutation, complementation studies with plasmids harboring wild-type MMR genes (*mutS* and *mutL*) were performed in mutator isolates from these three patients. In all cases the isolates were found to be defective in *mutS*. Thus, *mutS* was sequenced from the three mutator isolates and representative nonmutator isolates. Surprisingly, the three mutator isolates had the same inactivating mutation in *mutS* (4 bp deletion from nt 814), obviously absent in the nonmutator isolates. This specific mutation has not been previously noted in dozens of ofmutator variants sequenced so far [13], [14], [16], [38], [39], and is not reasonable to believe that it might have emerged independently in three different occasions. Therefore, these results provide evidence for the first time of interpatient spread of mutators. Moreover, they demonstrate the interpatient spread of a mutator lineage (ST-1089) evolved from a CF epidemic strain (ST-274).

### Interplay between Clonal Lineages, Mutator Phenotypes, Antimicrobial Susceptibility and Resistance Mechanisms

**Figure 2:**
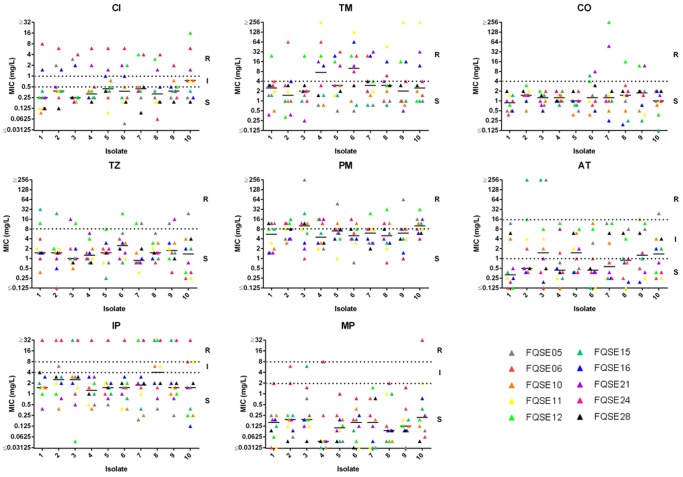
Evolution of minimal inhibitory concentrations (MICs) from the first to the last studied isolate from each patient. Each color represents a different patient. CI, ciprofloxacin; TM, tobramycin; CO, colistin; TZ, ceftazidime; PM, cefepime; AT, aztreonam; IP, impenem; MP, meropenem.

The overall susceptibility data for the collection of 100 isolates to 8 antipseudomonal agents is summarized in Table 2. Lowest susceptibility was observed for aztreonam (60% S) and highest for meropenem (96% S). However, resistance rates were highest for cefepime (30% R), tobramycin (30% R) and ciprofloxacin (24% R) and lowest for meropenem (1% R), aztreonam (4% R), and colistin (7% R). A significant trend towards increased MICs overtime to individual antibiotics was not noted, although all colistin resistant isolates occurred in the second half of the study (Figure 2). The low percentages of Aztreonam resistance might be of particular interest, considering its recent introduction for the treatment of CF chronic lung infection as inhaled therapy [40]. It should also be noted that EUCAST considers *P. aeruginosa* intrinsically nonsusceptible to this antibiotic (mainly due to the basal expression of MexAB-OprM efflux pump and pharmacokinetic/pharmacodynamic issues) (http://www.eucast.org/clinical_breakpoints/). Therefore, the percentage (60%) of susceptible isolates documented actually reflects the high number of hypersusceptible (MIC ranges 0.125–1 mg/L) isolates falling outside of wild-type MICs (2–16 mg/L) distributions (http://www.eucast.org/mic_distributions/). In addition of aztreonam, an important number of isolates showed hypersusceptibility to meropenem with MICs (<0.06 mg/L) falling outside of wild-type distributions.

**Table 2:** Antimicrobial susceptibility of the studied *P. aeruginosa* isolates.

Antibiotic	No. of isolates (n = 100)			No. of patients (n = 10)	
	S^a^	I^a^	R^a^	I+R^a^	R^a^
Ceftazidime	89	NA^b^	11	4	4
Cefepime	70	NA	30	8	8
Imipenem	79	5	16	7	4
Meropenem	96	3	1	3	1
Aztreonam	60	36	4	10	2
Ciprofloxacin	69	7	24	8	5
Tobramycin	70	NA	30	6	6
Colistin	93	NA	7	3	3

Resistance profiles (I+R) to the 8 antipseudomonal agents and mutator phenotypes for the 100 isolates are also indicated in Figure 1. Although the resistance profiles were variable within and across patients along the study period, a significant trend towards the accumulation of resistances was noted, increasing from an average of resistance to 1.1±1.2 antibiotics in the first isolate of each patient to 2.5±0.85 in the last isolate (paired t test, p = 0.016).Consistently with previous data [10], [11], [15], 29% of the isolates showed mutator phenotypes and 6 of the 10 patients showed at least 1 mutator isolate. In two patients all isolates were mutators and in other two, mutators emerged at late stages of colonization. In one more patient a mutator lineage emerged but it was not fixed and in other one it was replaced by the LES-1 epidemic strain (Figure 1). As described above, mutator variants were detected in 3 of the 4 patients colonized by epidemic clon A (ST-274/ST-1089). Mutator variants have also been previously detected in a small proportion of isolates of the LES-1 epidemic strain, but interpatient spread was not previously evidenced [24], [25]. In agreement with previous findings [10], [11], [19], [20], [41] a significant trend (p = 0.009) towards resistance to a higher number of antibiotics among mutator isolates (2.28±0.22) than among nonmutator isolates (1.49±0.17) was also noted, although not all mutator isolates were resistant and some nonmutator isolates, particularly noteworthy the LES-1 epidemic strain ST-146, were resistant to multiple antibiotics (Figure 1).

Resistance mechanisms [efflux pump (*mexB, mexD, mexF*, and *mexY*) and *ampC* overexpression and OprD production] were evaluated in the first and last isolate from each patient and clone and results are shown in Table 3. A trend towards accumulation of resistance mechanisms was noted, from 1.4±0.58 in the first to 2.1±0.88 in the last isolates, although the differences did not reach statistical significance (p = 0.06). The most frequent mechanism was *mexY* overexpression, documented in all 10 patients. Moreover, this mechanism was present in most patients (8 of 10) already in the early isolates (Table 3). The overexpression of the other efflux pumps was far more infrequent; *mexF* was documented in 3 patients, *mexD* in 2 and *mexB* only in one. AmpC overexpression was evidenced in 6 of the patients and lack of OprD production in the 4 patients colonized by imipenem resistant strains. Although a certain correlation was documented between *ampC* overexpression and ceftazidime resistance and lack of OprD and imipenem resistance, as previously observed [42], [43], a correlation between phenotype and genotype was not always evident, particularly concerning efflux pumps overexpression. Previously described efflux unbalance in CF [44] and antagonistic interactions between certain resistance mechanisms [45] could explain these discrepancies. Particularly, the previously documented frequent inactivation of the constitutive MexAB efflux system could well be responsible of the frequently observed aztreonam and meropenem hypersusceptibily (Table 3, Figure 2). The clone associated with a higher number of resistance mechanisms was ST-146/LES-1 previously associated with MDR phenotypes [43]. The initial MDR isolate from this clone already expressed 4 resistance mechanisms (MexY, MexF, MexD and OprD). The last isolate also expressed 4 resistance mechanisms (MexY, MexD, MexF, and OprD), but showed a significant reduction of the MDR profile, likely influenced by the modification of the mechanisms expressed (MexD instead of AmpC). Although not particularly associated with a high number of resistance mechanisms, all early and late isolates from the epidemic clone FQSE-A (ST-274/ST-1089) overexpressed *mexY.* Thus, *mexZ* was sequenced in all strains that overexpressed *mexY*, in order to determine the underlying genetic mechanism of resistance and to use *mexZ* mutations as epidemiological marker. As expected [46] most of the strains overexpressing *mexY* showed *mexZ* mutations, including deletions, premature stop codons, insertion sequences (IS), or nonsynonymous substitutions (Table 3). Remarkably, clone FQSE-A (ST-274/ST-1089) isolates from the different patients showed different *mexZ* mutations, denoting that interpatient spread precedes resistance development, except for the ST-274/ST-1089 *mutS* deficient mutator lineages (Table 3). Indeed, the ST-274/ST-1089 *mutS* deficient isolates showed the same *mexZ* mutation, demonstrating that the interpatient spread of the mutator lineages occurred after the acquisition of the resistance mechanism.

**Table 3:** Antimicrobial susceptibility results and resistant mechanisms detected in first and last studied isolates from each patient and clone.

Patient-Isolate	PFGEClone	ST	Mutator(Y/N)	MIC (mg/L)								Resistancemechanisms	*mexZ*mutations^a^
				TZ	PM	AT	IP	MP	CI	TO	CO		
FQSE06-0403	FQSE-A	274	N	0.064	1.5	0.125	1.5	0.016	0.125	4	0.38	*mexY*	S9P
FQSE06-1104	FQSE-A	274	Y	0.094	1	0.094	1	0.25	0.25	1	2	*mexY*	A194P
FQSE06-0610	FQSE-A	274	N	1.5	8	0.25	8	0.064	0.75	1.5	2	*mexY,* OprD-	Nt_292_Δ11
FQSE10-0503	FQSE-A	274	N	0.38	2	4	1.5	0.25	0.094	0.5	0.5	*mexY*	IS b
FQSE10-0111	FQSE-A	274	N	3	12	4	0.25	0.125	0.75	2	0.75	*ampC, mexY*	IS
FQSE15-0803	FQSE-A	274	N	1	8	0.38	2	0.125	0.38	1.5	1.5	*mexY*	A144V
FQSE15-0110	FQSE-A	1089	Y	1.5	12	2	0.38	0.38	0.38	1	0.016	*mexY*	A194P
FQSE24-0304	FQSE-A	1089	Y	1	12	0.19	>32	2	8	3	1	*mexY,*OprD-	A194P
FQSE24-1010	FQSE-A	1089	Y	0.38	4	0.38	>32	>32	6	4	1	*mexY,*OprD-	A194P
FQSE12-0603	FQSE-C	299	N	0.75	8	0.125	1	0.064	0.25	24	0.75	*mexY*	R125P
FQSE12-1206	FQSE-C	299	N	8	8	8	2	0.5	2	24	4	*ampC, mexY*	R125P
FQSE12-1007	FQSE-B	146	N	24	24	12	>32	1.5	4	4	>256	*ampC, mexY, mexF,* OprD-	Q164X
FQSE12-1110	FQSE-B	146	N	1	32	0.38	0.25	0.19	16	1	0.38	*mexY, mexF, mexD,*OprD-	Q164X
FQSE05-0403	FQSE-E	1108	N	16	12	12	1.5	0.25	0.25	1.5	0.5	*ampC*	WT
FQSE05-0704	FQSE-D	299	N	2	>256	>256	3	0.75	3	1.5	0.75	*ampC, mexY*	ND
FQSE05-0305	FQSE-F	1072	N	1	2	0.75	0.5	0.047	0.25	0.75	2	*mexY, mexF*	W185X
FQSE05-0807	FQSE-G	155	N	0.75	0.75	3	1.5	0.19	0.047	0.5	1.5	–	ND
FQSE05-0111	FQSE-E	1108	N	12	16	24	1.5	0.094	0.25	1	1	*ampC, mexY*	V43G
FQSE21-1003	FQSE-H	1088	N	0.5	1.5	0.5	0.38	0.19	0.25	0.38	1	*ampC, mexY*	Nt_61_Δ15
FQSE21-0505	FQSE-I	1109	N	16	16	8	3	0.5	2	16	2	*mexB, mexY*	K131R
FQSE21-0410	FQSE-H	1088	N	1.5	8	0.38	0.75	0.094	0.38	0.5	12	*mexY*	Nt_61_Δ15
FQSE21-1110	FQSE-I	1109	Y	8	16	0.75	1.5	0.25	1.5	32	1.5	*mexB, mexY*	K131R
FQSE28-1006	FQSE-J	1071	N	1.5	12	6	4	0.094	0.125	3	2	*ampC, mexY*	Nt_189_Δ12
FQSE28-1110	FQSE-J	1071	N	1	6	4	2	0.047	0.19	3	2	*ampC, mexY*	Nt_189_Δ12
FQSE11-0603	FQSE-K	701	N	1	3	0.25	1.5	0.032	0.125	2	1.5	*mexY*	WT
FQSE11-0608	FQSE-L	254	N	1	8	0.125	1	0.023	0.38	2	1.5	*mexY*	Nt_279_Δ12
FQSE11-1010	FQSE-K	701	N	2	8	0.25	8	2	0.75	>256	1	*mexY,* OprD-	WT
FQSE16-0803	FQSE-M	1073	Y	1.5	1.5	0.25	3	0.19	1.5	3	0.5	*mexF, mexD*	WT
FQSE16-0910	FQSE-M	1073	Y	3	6	2	0.125	0.75	0.25	12	2	*ampC, mexY, mexF*	R125P

a PAO1 and PA14 were used as reference wild-types sequences (www.pseudomonas.com).

b 1.2 Kb IS located in *mexX-mexZ* intergenic region (nt -72 respect *mexZ* coding sequence). Encodes a putative transposase identical to that previously reported in *Pseudomonas pseudoalcaligenes* CECT 5344 (ref ZP_10763279.1).

In summary, the presented results provide evidence for the interpatient spread of the CF epidemic strain ST-274. Much more importantly, they strongly suggest that ST-274 bacterial populations spreading among different patients were not a single genotype, but rather included a *mutS* deficient subpopulation that had already evolved into the new mutator lineage ST-1089 and had acquired specific resistance mutations. In other words, presented results provide the first evidence of interpatient dissemination of mutator lineages and denote their potential for unexpected short-term sequence type evolution (leading to MLST vs PFGE discrepancies), illustrating the complexity of *P. aeruginosa* population biology in CF.

## Materials and Methods

### Ethics Statement

This study was approved by the Research Committee of Hospital Son Espases. All clinical isolates used were obtained from a preexisting collection recovered over years from routine cultures and the study does not include patients` data.

### Clinical Isolates and Susceptibility Testing

The collection studied included 10 sequential *P. aeruginosa* isolates from each of 10 CF patients attended at hospital Son Espases, reference hospital of the Balearic Islands, Spain. Each of the sequential isolates included were separated by at least a 6-month interval, covering up to an 8-year period from 2003 to 2010. The first available isolate and the last available isolate (when the project was initiated) from each of the patients was always included in the studied collection. PAO1 strain was used as reference. The antibiotic susceptibility profiles (ceftazidime, cefepime, aztreonam, imipenem, meropenem, ciprofloxacin, tobramycin and colistin) were determined by Etest, using EUCAST breakpoints (http://www.eucast.org/).

### Molecular Typing

Clonal relatedness was evaluated in all isolates by PFGE. For this purpose, bacterial DNA embedded in agarose plugs prepared as described previously [47] was digested with SpeI. DNA separation was then performed in a contour-clamped homogeneous-electric-field DRIII apparatus (Bio-Rad, La Jolla, CA) under the following conditions: 6 V/cm^2^ for 26 h with pulse times of 5 to 40 s. DNA macrorestriction patterns were interpreted according to the criteria established by Tenover et al. [48]. Representative isolates from each clone and patient were further analyzed by MLST using available protocols and databases (http://pubmlst.org/paeruginosa/).

### Characterization of Resistance Mechanisms

The levels of expression of *ampC*, *mexB*,mexD, *mexF* and *mexY* were determined by Real-time reverse transcription (RT)-PCR according to previously described protocols [49], [50]. Briefly, strains were grown in 10 ml of LB broth at 37°C and 180 rpm to the late log phase (optical density at 600 nm [OD_600_] of 1) and collected by centrifugation. Total RNA was isolated by using the RNeasy minikit (Qiagen), dissolved in water, and treated with 2 U of Turbo DNase (Ambion) for 30 min at 37°C to remove contaminating DNA. The reaction was stopped by the addition of 5 µl of DNase inactivation reagent to the mixture. A 50-ng sample of purified RNA was then used for one-step reverse transcription and real-time PCR amplification using the Quanti Tect SYBR green RT-PCR kit (Qiagen) with a SmartCycler II instrument (Cepheid). Previously described primers [49], [50] were used for the amplification of *ampC*, *mexB*, *mexD*, *mexF*, *mexY*, and *rpsL* (used as a reference to normalize the relative amount of mRNA). Strains were considered positive for *ampC*, *mexD*, *mexF* or *mexY* overexpression when the corresponding mRNA level was at least 10-fold higher than that of PAO1, negative if lower than 5-fold, and borderline if between 5- and 10-fold. Strains were considered positive for *mexB* overexpression when the corresponding mRNA level was at least 3-fold higher than that of PAO1, negative if lower than 2-fold and borderline if between 2- and 3-fold. All PCRs were performed in duplicate. Mean values (± standard deviations) of mRNA levels obtained in three independent duplicate experiments were considered. Previously characterized strains overexpressing these mechanisms were used as controls [50]. Additionally, the gene encoding the transcriptional regulator of MexXY-OprM efflux pump, *mexZ,* was fully sequenced in representative isolates, from each patient and clone, showing *mexY* overexpression [32]. After duplicate PCR amplification, sequencing reactions were performed with the Big Dye Terminator kit (PE Applied Biosystems, Foster City, CA), and sequences were analyzed on an ABI Prism 3100 DNA sequencer (PE Applied Biosystems). The resulting sequences were then compared with that of wild-type PAO1 and those available at GenBank. Finally, outer membrane protein (OMP) profiles were analyzed by sodium dodecyl sulfate-polyacrylamide gel electrophoresis (SDS-PAGE) and stained with Coomassie blue following previously described protocols [45]. Obtained OprD profiles were compared with those of PAO1 and its OprD-deficient mutant.

### Mutant Frequencies and Genetic Basis of Hypermutation

Rifampicin (300 mg/L) resistance mutant frequencies were determined in all strains following previously established procedures [10]. To explore the genetic basis for the mutator phenotypes, complementation studies were performed as described previously [15]. Briefly, plasmid pUCPMSharbouring PAO1 wild-type *mutS*, plasmid pUCPML harbouring PAO1 wild-type *mutL*, and plasmid pUCP24, a control cloning vector, were electroporated into the mutator isolates. Complementation was demonstrated by reversion of the increased rifampicin resistance mutant frequencies in two independent transformant colonies for each strain. Previously described primers and protocols [15] were used for the amplification and sequencing of *mutS* or *mutL* genes according to the results of complementation experiments.

### Statistical Analysis

The Graph Pad Prism 5 software was used for graphical representation and statistical analysis. Quantitative variables were compared using the Mann-Whitney U-test or the Student’s *t* test as appropriate. Categorical variables were compared using the χ^2^ test. A *p* value of less than 0.05 was considered statistically significant.
